# Smoking-attributable morbidity: acute care hospital diagnoses and days of treatment in Canada, 2002

**DOI:** 10.1186/1471-2458-7-247

**Published:** 2007-09-18

**Authors:** Dolly Baliunas, Jayadeep Patra, Jürgen Rehm, Svetlana Popova, Benjamin Taylor

**Affiliations:** 1 Public Health Sciences Department, University of Toronto, Toronto, Canada; 2 Centre for Addiction and Mental Health, Toronto, Canada; 3 Research Institute for Public Health and Addiction, Zurich, Switzerland

## Abstract

**Background:**

Smoking is one of the most important risk factors for burden of disease. Our objective was to estimate the number of hospital diagnoses and days of treatment attributable to smoking for Canada, 2002.

**Methods:**

Distribution of exposure was taken from a major national survey of Canada, the Canadian Community Health Survey. For chronic diseases, risk relations were taken from the published literature and combined with exposure to calculate age- and sex-specific smoking-attributable fractions (SAFs). For fire deaths, SAFs were taken directly from available statistics. Information on morbidity, with cause of illness coded according to the International Classification of Diseases version 10, was obtained from the Canadian Institute for Health Information.

**Results:**

For Canada in 2002, 339,179 of all hospital diagnoses were estimated to be attributable to smoking and 2,210,155 acute care hospital days. Ischaemic heart disease was the largest single category in terms of hospital days accounting for 21 percent, followed by lung cancer at 9 percent. Smoking-attributable acute care hospital days cost over $2.5 billion in Canada in 2002.

**Conclusion:**

Since the last major project produced estimates of this type, the rate of hospital days per 100,000 population has decreased by 33.8 percent. Several possible factors may have contributed to the decline in the rate of smoking-attributable hospital days: a drop in smoking prevalence, a decline in overall hospital days, and a shift in distribution of disease categories. Smoking remains a significant health, social, and economic burden in Canada.

## Background

Smoking is responsible for high levels of morbidity and mortality. Smoking causes substantially increased risk of lung cancer, upper aerodigestive cancer, several other cancers, heart disease, stroke, chronic respiratory disease and a range of other medical conditions[1]. In 1996, a major study estimating the economic costs attributable to substance abuse in Canada (1992 data), was published[2]. As part of the analysis, the numbers of hospital separations and days of hospital treatment that could be attributed to smoking tobacco were estimated to be 208,095 and 3,024,265 respectively. This paper updates the estimates for the year 2002, using age- and sex-specific relative risks from recent meta-analyses and morbidity classified according to the International Classification of Disease version 10 (ICD-10).

## Methods

The aim of the present study was to estimate the proportion of acute care hospital diagnoses and hospital days attributable to smoking in Canada for the year 2002.

### Identification of diseases and meta-analyses on smoking risk relations

To identify the malignant and non-malignant health conditions for inclusion in the estimate, this analysis was guided by the 2004 Health Consequences of Smoking: A Report from the Surgeon General[1] which considers the following criteria in judgments of causality: consistency, strength of association, specificity, temporality, coherence, dose-response, and experimental evidence. The 2004 Surgeon General's report implemented a standardized, hierarchical language to summarize conclusions about causality, the highest of which is, "evidence is sufficient to infer causality". Only health outcomes for which the previous conclusion was reached for active smoking were included in the present analysis. Additionally, two conditions for which the causal role of passive smoking is conclusive were included.

Once identified, conditions were translated into corresponding ICD-10 codes. Finally, a comprehensive search strategy of current meta-analyses was performed for each disease category and its risk relationship with smoking.

Meta-analyses were identified using the Pubmed and OVID (1966 – January week 3, 2005) databases. Search criteria were: smoking or tobacco, meta-analysis, and each disease category described in this paper.

Meta-analyses that included measures of smoking dose were preferred over those that only used current/former/never categories. However, if relative risks (RRs) for dose-response specific exposures were not found among the meta-analyses, current/former/never or ever/never categories were used. In 16 out of 20 disease categories included for active smokers, dose-response specific RRs were available. In two categories, plus one for smokers aged 65 or older, only current/former/never RRs were available. In two categories, only ever/never RRs were available. Similarly, analyses that included age- and sex-stratified estimates of relative risk were preferred over more crude estimates. Age-specific estimates were available for ischaemic heart disease (IHD), cardiac arrhythmias, heart failure, and cerebrovascular disease. In cases where a more recently published meta-analysis did not exist, the relative risk from English et al [3] was used. When a meta-analysis was published later than 1995, there was usually only one that presented data on smoking dose, so it was used as the source of relative risk. If there was more than one, all were examined, and that of highest quality and the most comprehensive based on age and smoking dose categories was chosen.

The ICD-10 codes of all disease categories related to smoking included in the present analysis are presented in Table 1.

**Table 1:** Smoking related disease categories and sources of risk relations

**Condition**	**ICD-10 code**	**Source from meta-analysis or SAF**
Mental and behavioural disorders due to use of tobacco [tobacco abuse]	F17	100% SAF per definition
Toxic effect of tobacco and nicotine	T65.2	100% SAF per definition
Oropharyngeal cancer	C00-C14, D00.0	English et al., 1995 [3]
Oesophageal cancer	C15, D00.1	English et al., 1995 [3]
Stomach cancer	C16, D00.2	Tredaniel et al., 1997 [15]
Pancreatic cancer	C25, D01.9	English et al., 1995 [3]
Laryngeal cancer	C32, D02.0	English et al., 1995 [3]
Trachea, bronchus and lung cancers	C33-C34	Simonato et al., 2001 [16]
Cervical cancer	C53, D06	Plummer et al., 2003 [17]
Urinary tract cancer	C64-C68	Zeegers et al., 2000 [18]
Renal cell carcinoma	C64	Hunt, 2005 [19]
Bladder cancer	C67, D09.0	Brennan et al., 2000; 2001 [20,21]
Acute myeloid leukaemia	C92.0	Brownson et al., 1993 [22]
Ischaemic heart disease	I20-I25	Law, 1997 & Law, 2003 [23,24]
Pulmonary circulatory disease	I26-I28	English et al., 1995 [3]
Cardiac arrhythmias	I47-I49	Follow IHD
Heart failure; complications and ill-defined descriptions disease	I50-I51	Follow IHD
Cerebrovascular diseases	I60-I69	English et al., 1995 [3]
Atherosclerosis	I70-I79	English et al., 1995 [3]
Pneumonia & influenza	J10-J18	English et al, 1995 [3]
Chronic obstructive pulmonary disease	J40-J44	Single et al., 1996 [2]
Ulcers	K25-K28	English et al., 1995 [3]
Low birth weight and short gestation	P05-P07	English et al., 1995 [3]
Sudden infant death syndrome	R95	English et al., 1995 [3]
Fires	X00-X09	Council of Canadian Fire Marshals and Fire Commissioners. Annual Report 2000, 2003 [9]

Passive smoking-attributable morbidity was derived by applying sex-specific RRs and rates of morbidity from lung cancer and age- and sex-specific RRs and rates of morbidity from IHD to the population of Canadians who have never smoked but are exposed to environmental tobacco smoke (ETS) in the home from spouses and other sources. RR estimates were obtained from the most comprehensive meta-analyses applicable to Canada. This procedure did not include the effect of ETS exposure on current smokers and thus the total burden of smoking was underestimated.

All RRs used may be obtained from the comprehensive report of this cost study [4].

### Prevalence of smoking in Canada

Smoking prevalence for different levels of smoking consumption for Canada as a whole were obtained from the Canadian Community Health Survey 2003 (CCHS cycle 2.1) [5], a population based representative survey conducted by Statistics Canada. All prevalence estimates were sex- and age group-specific. The categorizations of smoking status, however, varied by disease based on the RRs available in the meta-analyses; for example, for COPD, RRs risks were available for current, former and never smokers, thus the prevalence of current, former and never smokers was used. For each disease for which the identified meta-analysis included dose-response-specific RRs, prevalence estimates also were dose-specific (e.g., never, former, current, 1–14, 15–24, 25+ cigarettes per day). Current smokers, those who reported occasional smoking or daily smoking, were further categorized by number of cigarettes per day when sufficient information existed to do so.

The prevalence of non-smokers living inside a home where another person smokes was also available from the CCHS data set and was used to calculate 2002 passive smoking hospital diagnoses and days.

The sample weights provided by Statistics Canada to ensure comparability between the CCHS sample and the Canadian population were used to calculate prevalence based on sex and age groups. The age groups used were 15–29, 30–44, 45–59, 60–69, 70–79, and 80+.

### Morbidity data

Acute care hospital diagnoses and days data in Canada for 2002 were obtained from the Canadian Institute for Health Information (CIHI) on national and provincial levels according to ICD-10. For the national level, data were provided for each disease condition as well as for each sex and five-year age group from 0 to 80. However, the national level data were actually composed of only seven provinces and two territories (Alberta, British Columbia, Newfoundland, Northwest Territories, Nova Scotia, Ontario, Prince Edward Island, Saskatchewan, Yukon Territory). Based on these, data for Canada as a whole were estimated using the total population: the disease-specific rate of occurrence observed in the data provided was applied to the total population of Canada to obtain the estimated number of disease-specific occurrences.

The Hospital Morbidity Database (HMDB) held by CIHI captures information on patients separated (through discharge or death) from acute care facilities in Canada. As such, it provides national data on acute care hospitalizations by diagnoses and procedures. Day procedures (e.g., day surgeries), outpatient, and emergency department visits are not captured in this database. Stillborns and cadaveric donor "discharges" are excluded, whereas newborns are included in the HMBD database. Figures are based on facility geography, that is, where the hospital is located (i.e., it may include non-Canadians). Additionally, the statistics reflect the number of hospitalizations, which is somewhat higher than the number of individuals diagnosed since individuals with multiple admissions during a single year would be counted more than once.

Hospital days (Length of Stay) is associated with the condition coded as Most Responsible Diagnosis (MRD) on the patient's hospital record. In other words, the MRD accounts for the majority of a patient's days in hospital. MRD is the one diagnosis that describes the most significant condition of the patient that is responsible for his/her stay in hospital. In a case where multiple diagnoses may be classified as most responsible, coders are instructed to code the diagnosis responsible for the longest length of stay[6]. However, there is still overlap in the database, i.e. more than one diagnosis listed per hospital stay. Thus, to calculate hospital days attributable to tobacco smoking without overlap, the disease-specific hospital days were scaled down by the factor of overall hospital days in Canada divided by the total of the disease-specific hospital days.

### Computing smoking-attributable fractions

The smoking-attributable fraction (SAF) is defined as the fraction of the disease in the population that would not have occurred if the effect associated with smoking was absent [7,8]. SAFs were assessed for different specific causes of illnesses by two methods:

• chronic disease SAFs were calculated by combining exposure from CCHS and RR estimates from meta-analyses,

• fire injury was calculated using direct estimates of smoking involvement from the Council of Canadian Fire Marshals and Fire Commissioners[9].

We used the most comprehensive meta-analysis for each condition, as described above (see also Table 1). The RR for each condition was combined with different levels of smoking consumption for each sex and age group and an attributable fraction was obtained using the following formula (see Walter, 1976, 1980).

SAF=∑i=1kPi(RRi−1)∑i=0kPi(RRi−1)+1
 MathType@MTEF@5@5@+=feaafiart1ev1aaatCvAUfKttLearuWrP9MDH5MBPbIqV92AaeXatLxBI9gBaebbnrfifHhDYfgasaacH8akY=wiFfYdH8Gipec8Eeeu0xXdbba9frFj0=OqFfea0dXdd9vqai=hGuQ8kuc9pgc9s8qqaq=dirpe0xb9q8qiLsFr0=vr0=vr0dc8meaabaqaciaacaGaaeqabaqabeGadaaakeaacqWGtbWucqWGbbqqcqWGgbGrcqGH9aqpdaWcaaqaamaaqahabaGaemiuaa1aaSbaaSqaaiabdMgaPbqabaGcdaqadaqaaiabdkfasjabdkfasnaaBaaaleaacqWGPbqAaeqaaOGaeyOeI0IaeGymaedacaGLOaGaayzkaaaaleaacqWGPbqAcqGH9aqpcqaIXaqmaeaacqWGRbWAa0GaeyyeIuoaaOqaamaaqahabaGaemiuaa1aaSbaaSqaaiabdMgaPbqabaGcdaqadaqaaiabdkfasjabdkfasnaaBaaaleaacqWGPbqAaeqaaOGaeyOeI0IaeGymaedacaGLOaGaayzkaaGaey4kaSIaeGymaedaleaacqWGPbqAcqGH9aqpcqaIWaamaeaacqWGRbWAa0GaeyyeIuoaaaaaaa@54E5@

i: exposure category with baseline exposure or no smoking i = 0.

RR_i_: relative risk at exposure level i compared to no consumption.

P_i_: prevalence of the i*th *category of exposure.

The SAFs were then applied to the hospital diagnosis and length of stay information to estimate the smoking-attributable morbidity by age and sex. To illustrate this procedure, a simple example follows. For chronic obstructive pulmonary disorder (COPD) the all-age and all-sex RRs for current smokers, and former smokers, relative to never smokers are 9.80, 6.70. The prevalence, with rounding error, of current, former, and never smokers among men aged 45–59 were 26.0, 53.0, and 21.1. Simple algebra produces a SAF of 0.84, which is applied to the 978 hospital diagnoses of COPD among all Canadian men of this age group to determine that 823 of the diagnoses were attributable to smoking (355 and 468 among the current and former smokers respectively). It is notable that although the RR among current smokers is greater than that among former smokers, the greater prevalence of former smokers among men aged 45–59 resulted in a larger smoking-attributable burden of COPD among the former smokers.

### Estimating costs of acute care hospital days

Daily average per-capita cost for acute care hospital separations for each available province and territory was obtained from CIHI [10]. For Nunavut, average per-capita cost was not available; therefore, average per-capita cost for Northwest Territories was substituted. To obtain the total costs for each province and territory, daily average per-capita cost was applied to the total number of smoking-attributable acute care hospital days. These numbers were summed to obtain the total for Canada. A similar procedure was followed for those acute care hospital days attributable to alcohol and illegal drugs for comparison purposes.

## Results

Table 2 gives an overview of the estimated smoking exposure in Canada by sex and age group. As expected, men smoked more than women on average, and smoking prevalence decreased with age.

**Table 2:** Smoking status by sex and age group in Canada, 2002

**Smoking categories**		**15–29 years**	**30–44 years**	**45–59 years**	**60–69 years**	**70–79 years**	**80+ years**	**Overall (All ages)**
Current	Female	26.3	24.7	23.0	15.1	10.3	5.9	**21.8**
	Male	31.0	30.7	26.0	17.2	9.8	7.5	**26.3**
Former	Female	26.2	38.3	43.5	45.2	42.7	38.9	**37.8**
	Male	25.8	38.9	53.0	64.2	70.4	73.5	**44.6**
Never	Female	47.5	36.9	33.4	39.8	47.1	55.2	**40.4**
	Male	43.1	30.4	21.1	18.7	19.7	19.0	**29.1**
**Total per sex**		**100**	**100**	**100**	**100**	**100**	**100**	**100**

Source: CCHS cycle 2.1 (2003)

Table 3 provides the estimates of smoking-attributable hospital diagnoses. Overall, in Canada in 2002, 339,179 hospital diagnoses from acute care facilities were smoking-attributable, accounting for 218,791 hospital diagnoses among men and 120,389 among women. Please note: these numbers were derived by multiplying SAFs with the number of diagnoses for each category, thereby producing numbers with decimals. As a result, there may be rounding errors after collapsing numbers over different categories. Among hospital diagnoses caused by smoking, the three biggest contributors were cardiovascular disease, malignant neoplasms and respiratory disease. Indeed, the single disease category of ischaemic heart disease accounted for 30.3 percent of the smoking-attributable hospital diagnoses (102,704 diagnoses; males: 75,814; females: 26,890). The next largest single categories were cardiac arrythmias (29,623 hospital diagnoses, males: 19,222, females: 10,401), and lung cancer (28,929 hospital diagnoses, males: 18,488, females: 10,441). With respect to age, the overall average age for a smoking-attributable hospital diagnosis was 61.9 years for men and 62.8 years for women. There were notable differences between disease categories. For fire injuries, the average age for a smoking-attributable hospital diagnosis was 44.9 years for men and 53.9 years for women. Smoking-attributable hospital diagnoses due to the toxic effects of tobacco and nicotine occurred at the age of 29.5 years for men and 37.0 years for women.

**Table 3:** SAFs*, hospital diagnoses and acute care hospital days attributable to smoking for major disease categories in Canada, 2002

*Disease condition (for definition by ICD 10 see Table 1)*	***Number of diagnoses***							***Hospital days***				
	***SAF in % (all ages)***		***mean age at diagnosis***		***Smoking-attributable diagnoses***			***SAF in % (all ages)***		***Smoking-attributable hospital days***		

	***M***	***F***	***M***	***F***	***M***	***F***	***Overall***	***M***	***F***	***M***	***F***	***Overall***

**ACTIVE SMOKERS**												
Malignant neoplasms												
Oropharyngeal cancer	58.7	51.3	59.6	60.1	2,128	1,116	3,244	34.8	30.2	13,302	6,125	19,427
Oesophageal cancer	48.8	40.0	66.4	68.8	1,338	387	1,725	29.4	23.6	9,453	3,140	12,593
Stomach Cancer	16.4	12.5	67.0	66.1	546	233	780	9.8	7.4	4,308	2,068	6,376
Pancreatic cancer	17.7	13.4	65.6	67.5	623	478	1,101	10.4	8.0	4,189	3,831	8,021
Laryngeal cancer	67.7	60.8	65.8	64.2	950	236	1,187	40.4	35.6	7,637	1,815	9,452
Lung cancer	88.6	63.8	68.6	66.2	18,488	10,441	28,929	53.4	37.9	124,082	75,280	199,362
Cervical cancer	--	36.4	--	48.5	--	1,381	1,381	--	21.4	--	4,807	4,807
Urinary tract	55.6	38.3	68.7	68.2	8,336	2,392	10,728	33.2	22.6	38,017	13,149	51,165
Renal cell carcinoma	26.0	7.5	63.7	62.0	993	176	1,169	15.7	4.2	5,996	1,116	7,112
Bladder cancer	67.7	52.4	72.5	72.6	7,336	1,832	9,169	40.9	31.3	29,288	8,604	37,892
Acute myeloid leukemia	21.9	17.7	59.2	56.4	465	310	775	12.8	10.7	4,461	3,316	7,777
**Total malignant neoplasms**					32,875	16,974	49,849			205,449	113,530	318,979
Tobacco abuse												
Tobacco abuse	100.0	100.0	55.6	52.9	4,042	3,481	7,522	100.0	100.0	18,204	15,776	33,979
Toxic effect of tobacco and nicotine	100.0	100.0	29.5	37.0	7	7	14	100.0	100.0	9	30	39
**Total tobacco abuse**					4,049	3,488	7,536			18,213	15,805	34,018
Cardiovascular diseases												
Ischaemic heart disease												
Age < 45 yrs	51.4	44.3	36.6	36.3	6,534	1,758	8,293	30.4	26.5	20,044	6,548	26,593
45–59 yrs	42.2	37.3	52.0	52.0	32,360	8,979	41,339	25.2	22.3	119,120	36,466	155,586
60–69 yrs	29.1	23.4	64.5	64.5	22,681	8,294	30,975	17.4	14.0	103,449	42,245	145,694
70–79 yrs	10.0	7.3	74.5	74.5	9,346	4,518	13,865	6.0	4.3	52,164	26,986	79,150
80+ yrs	8.9	5.1	87.0	87.0	4,891	3,340	8,232	5.3	3.1	32,243	24,358	56,601
Pulmonary circulatory disease	81.0	76.4	65.2	65.8	9,251	10,601	19,852	47.9	44.9	81,952	90,735	172,687
Cardiac arrythmias												
Age < 45 yrs	43.6	37.5	33.6	32.9	2,025	1,323	3,348	24.7	20.5	9,158	5,577	14,735
45–59 yrs	42.2	37.3	52.0	52.0	5,483	2,348	7,832	25.2	22.2	29,911	12,656	42,568
60–69 yrs	29.1	23.4	64.5	64.5	5,782	2,650	8,432	17.3	13.9	35,626	16,305	51,931
70–79 yrs	10.0	7.3	74.5	74.5	3,493	2,069	5,562	6.0	4.3	24,437	14,648	39,084
80+ yrs	8.9	5.1	87.0	87.0	2,439	2,011	4,450	5.3	3.1	19,254	16,975	36,228
Heart failure												
Age < 45 yrs	41.0	31.7	34.1	34.2	1,005	631	1,635	23.5	18.0	6,764	4,771	11,535
45–59 yrs	42.2	37.3	52.0	52.0	3,841	1,904	5,745	25.2	22.3	24,850	13,435	38,285
60–69 yrs	29.1	23.4	64.5	64.5	4,553	2,517	7,070	17.4	14.0	31,466	18,134	49,600
70–79 yrs	10.0	7.3	74.5	74.5	3,008	1,911	4,918	6.0	4.3	22,437	15,173	37,610
80+ yrs	8.9	5.1	87.0	87.0	2,748	2,319	5,066	5.3	3.1	22,183	20,520	42,703
Cerebrovascular diseases												
Age < 65 yrs	39.1	35.4	52.8	50.5	5,033	3,449	8,482	23.5	21.3	45,470	32,326	77,796
> = 65 yrs	14.7	10.5	76.9	78.4	5,535	4,113	9,647	8.8	6.2	58,485	48,105	106,589
Atherosclerosis	33.5	36.9	68.9	69.4	14,036	9,896	23,933	19.8	21.7	109,603	80,842	190,445
**Total cardiovascular diseases**			62.2	65.2	144,044	74,631	218,675			848,617	526,806	1,375,423
Respiratory diseases												
Pneumonia & Influenza	17.3	12.8	69.4	68.3	13,246	8,648	21,894	11.5	8.3	118,770	74,914	193,684
Chronic obstructive pulmonary disease	82.6	76.9	67.0	66.8	5,268	4,463	9,731	49.3	45.5	32,022	27,608	59,630
**Total respiratory diseases**			68.7	67.8	18,514	13,111	31,625			150,793	102,522	253,315
Intestinal diseases												
Ulcer	47.7	38.4	65.4	68.3	6,237	4,026	10,263	28.8	22.6	45,416	31,551	76,967
Perinatal period												
Low birthweight and short gestation	24.7	20.6	0.0	0.0	6,770	5,057	11,827	14.8	12.3	59,906	42,580	102,485
Sudden infant death syndrome	31.2	26.5	0.0	0.0	2	1	3	19.6	16.7	2	0	2
**Total perinatal diseases**			0.0	0.0	6,772	5,059	11,832			59,907	42,580	102,487
Injury												
Fire injury	24.6	23.4	44.9	53.9	244	101	346	24.6	24.6	1,529	737	2,266
Total active smokers			61.6	62.3	212,735	117,390	330,126			1,329,923	833,531	2,163,455
**PASSIVE SMOKERS**												
Lung cancer	1.6	1.4	66.5	65.2	330	231	561	0.9	0.8	2,163	1,643	3,806
Ischaemic heart disease	1.8	1.5	64.7	70.5	5,725	2,768	8,493	1.0	0.8	27,073	15,821	42,894
**Total passive smokers**			64.8	70.1	6,056	2,999	9,054			29,235	17,465	46,700
**TOTAL SMOKING-ATTRIBUTABLE**			61.9	62.8	218,791	120,389	339,179			1,359,159	850,996	2,210,155

* The SAF (smoking attributable fraction) is the fraction of the disease in the population that would not have occurred if the effect associated with smoking was absent. It is calculated by means of a formula that combines prevalence of smoking and relative risk of a particular disease. The SAF is then applied to the total number of occurrences of disease to estimate the number of occurrences that are attributable to smoking.

Table 3 also provides the estimates of smoking-attributable acute care hospital days. Overall, in Canada in 2002, 2,210,155 hospital days were estimated to be attributable to smoking (males: 1,359,159; females: 850,996). This accounts for 10.3 percent of all acute care hospital days in Canada in 2002. The vast majority (97.9 percent) of these hospital days were due to active, as opposed to passive, smoking. However, only two categories of disease were considered to have sufficient evidence to conclude a causal relationship with passive smoke exposure. As further research is generated, additional disease categories may be broadly recognized, and hence affect future estimates of smoking-attributable morbidity.

Among the acute care hospital days attributable to smoking, the three biggest contributors were cardiovascular disease, malignant neoplasms and respiratory disease. The single disease category that accounted for the most smoking-attributable hospital days was ischaemic heart disease (21.0%; 463,625 hospital days; males: 327,021; females: 136,604). The next largest single categories were lung cancer (9.0%; 199,362 hospital days, males: 124,082; females: 75,280), and pneumonia and influenza (8.8%; 193,684 hospital days; males: 118,770, females: 74,914).

Acute care hospital costs are shown in table 4.

**Table 4:** Costs of acute care hospital days attributable to smoking, alcohol consumption and illegal drug use in Canada, 2002

	Acute care hospital days costs (in millions dollars)
Smoking	2551.2
Alcohol consumption	1458.6
Illegal drug use	426.4

Source: Rehm J, Baliunas D, Brochu S, Fischer F, Gnam W, Patra J, Popova S, Sarnocinska-Hart A, Taylor B. The costs of substance abuse in Canada 2002. Canadian Centre on Substance Abuse. Ottawa, 2006. ISBN 1-896323-92-8

## Conclusion

Overall, there were 2,210,155 acute care hospital days attributable to smoking, accounting for 10.3 percent of all those in Canada in 2002. At the time of the last cost study (data for 1992) for which similar estimates were produced, the number of acute care hospital days attributed to smoking was higher at 3,024,265, which accounted for 7.3 percent of all acute care hospital days. In considering these numbers, it must be noted that the total number of hospital days due to overall morbidity has decreased due to changes in the Canadian health care system meant to reduce health care expenditures such as progress in treatment associated with a shorter length of stay, and a shift from inpatient to outpatient treatment; the total acute care hospital days decreased from approximately 41.5 million in 1992 to 21.5 million in 2002 [2]. In addition, the size of the population has increased from 28,366,737 in 1992 to 31,372,587 in 2002 [11]. Therefore, in order to get the best sense of the cost of smoking on hospital days, it is most appropriate to consider the rate of hospital days per 100,000 population which has decreased from 10,635 in 1992 to 7,045 in 2002, a decrease of 33.8 percent. Thus, there have been gains made in the health of Canadians with respect to the morbidity due to smoking since the last Canadian cost study.

There are several possible factors that have contributed to the decline of the rate of smoking-attributable hospital days. The proportion of Canadians who are current smokers has decreased. The prevalence of current smoking decreased from 31.1 percent in 1991 to 23.0 percent in 2003 [5,12]. It may be premature to conclude that the observed decreases in smoking-attributable hospital days have been caused by the decreases in smoking prevalence. Most diseases caused by smoking have a long latent period. Thus, declines in smoking in the last few years would not be expected to produce substantial improvements in the burden of smoking related diseases on the Canadian population for some time.

In addition to the decrease in smoking levels, there have been important shifts in the overall distribution of causes of death in Canada during this period. Mortality due to heart disease has been decreasing for several decades. From 1992 to 1999, the age-standardized mortality rate (standardized by the 1991 Canadian population) decreased from 272.2 to 232.9 per 100,000 population [13]. Ischaemic heart disease was the single greatest cause of smoking-attributable morbidity in both 1992 and 2002, but overall deaths due to ischaemic heart disease, not just those attributable to smoking, have decreased, accounting for 22.1 percent of all deaths in 1992 and 18.2 percent of all deaths in 2002. Morbidity data is not collected in the same manner as mortality data, however according to the Public Health Agency of Canada, among males, the age-standardized hospital separation rate for ischaemic heart disease (standardized by the 1991 Canadian population) decreased from 622.6 to 564.9 per 100,000 in 1999[13]. Further, it should be acknowledged that the decline in heart disease in Canada began prior to recently observed decreases in prevalence of smoking. Similarly, the proportion of smoking-attributable lung cancer hospital days has declined from 14.1 percent to 9.2 percent of the total smoking attributable hospital days. The overall incidence of lung cancer has decreased over recent years. In 1992 the age-standardized incidence rate of lung cancer among males was 90.2 per 100,000 population. By 2002, this had declined to 73.3 per 100,000 population. Among females, the incidence, though lower, has increased from 39.7 to 45.2 per 100,000 population.

Although the same approach was utilized in calculating the smoking-attributable fractions in both 1992 and 2002, different inputs were used. That is, the relative risks that were used to estimate the relationships between smoking and specific health outcomes were updated to reflect recently available literature. Also, this analysis, in determining which health outcomes to include in these estimates, was guided by the report of the Surgeon General and thus included acute myeloid leukaemia but did not include chemotherapy under a separate heading. This may be suspected of causing significant differences in the estimates for the two years, however in one of a number of sensitivity analyses published in a recent article, when 2002 data were analyzed using the 1992 approach in addition to our present approach the estimates were very similar (2,316,166 versus 2,210,155)[14].

The smoking-attributable hospital diagnoses estimates produced in this analysis cannot be compared with the first Canadian cost study, as that work measured hospital separations and not diagnoses.

The burden of smoking-attributable morbidity as measured by hospital diagnoses has decreased substantially since the last Canadian cost study, which estimated the burden of morbidity attributable to smoking in Canada. While this is reason for optimism, smoking continues to present a serious health concern in Canada; smoking causes substantial morbidity in this country, and carries huge humanitarian and financial costs. In 2002, smoking-attributable acute care hospital days alone cost Canadian taxpayers in excess of 2.5 billion dollars[4], far more than alcohol and illegal drug use consumption combined. Given concerns about the increasing cost of the Canadian health care system and the pressures to preserve this same system, here is a viable target for cost savings. Smoking-attributable morbidity is avoidable. Thus, it is vital that policy and policy-makers address this substantial health, social, and economic burden in Canada.

## Competing interests

The author(s) declare that they have no competing interests.

## Authors' contributions

DB selected the disease outcomes, collected the risk relation data for these outcomes, aided in the statistical analysis, and drafted the manuscript. JP carried out the statistical analysis. SP and BT participated in the collection of risk relation and morbidity data. JR conceived of the study, and participated in its design and coordination and helped to draft the manuscript. All authors read and approved the final manuscript.

## Pre-publication history

The pre-publication history for this paper can be accessed here:



## References

[B1] U.S.Department of Health and Human Services (2004). The health consequences of smoking: a report of the Surgeon General..

[B2] Single E, Robson L, Xie X, Rehm J (1996). The costs of substance abuse in Canada..

[B3] English DR, Holman CDJ, Milne E, Winter MJ, Hulse GK, Codde G, Bower CI, Corti B, de Klerk N, Knuiman MW, Kurinczukn JJ, Lewin GF, Ryan GA (1995). The quantification of drug caused morbidity and mortality in Australia 1995..

[B4] Rehm J, Baliunas D, Brochu S, Fischer B, Gnam W, Patra J, Popova S, Sarnocinska-Hart A, Taylor B (2006). The costs of substance abuse in Canada 2002..

[B5] Canada S (2003). Canadian Community Health Survey, Cycle 2.1 [Catalogue 82M0013XCB]..

[B6] CIHI - Canadian Institute for Health Information (2004). Data quality documentation: discharge abstract database 2002-2003..

[B7] Walter SD (1976). The estimation of interpretation of attributable risk in health research.. Biometrics.

[B8] Walter SD (1980). Prevention of multifactorial disease.. American Journal of Epidemiology.

[B9] Council of Canadian Fire Marshalls and Fire Commissioners (2003). Fire Losses in Canada Annual Report, 2000..

[B10] CIHI - Canadian Institute for Health Information (2004). Canadian MIS database: hospital finance performance indicators 1999-2000 to 2001-2002..

[B11] Canada S (2006). Estimates of population by age and sex for Canada, Provinces and Territories..

[B12] Canada S (1992). General Social Survey, Cycle 6 (1991)[catalogue 89F0115XIE]..

[B13] Canada PHA (2006). Cardiovascular disease surveillance on-line.. http://dsol-smed.phac-aspc.gc.ca/dsol-smed/cvd/index_e.html.

[B14] Patra J, Taylor B, Rehm J, Popova S, Baliunas D (2007). Substance-attributable morbidity and mortality changes to Canada's epidemiological profile: measurable differences over a ten-year period.. Canadian Journal of Public Health.

[B15] Tredaniel J, Boffetta P, Buiatti E, Saracci R, Hirsch A (1997). Tobacco smoking and gastric cancer: review and meta-analysis.. International Journal of Cancer.

[B16] Simonato L, Agudo A, Ahrens W, Benhamou E, Benhamou S, Boffetta P, Brennan P, Darby SC, Forastiere F, Fortes C, Gaborieau V, Gerken M, Gonzales CA, Jockel KH, Kreuzer M, Merletti F, Nyberg F, Pershagen G, Pohlabeln H, Rosch F, Whitley E, Wichmann HE, Zambon P (2001). Lung cancer and cigarette smoking in Europe: an update of risk estimates and an assessment of inter-country heterogeneity.. International Journal of Cancer.

[B17] Plummer M, Herrero R, Franceschi S, Meijer CJ, Snijders P, Bosch FX, de Sanjose S, Munoz N (2003). Smoking and cervical cancer: pooled analysis of the IARC multi-centric case-control study.. Cancer Causes & Control.

[B18] Zeegers MP, Tan FE, Dorant E, van Den Brandt PA (2000). The impact of characteristics of cigarette smoking on urinary tract cancer risk: a meta-analysis of epidemiologic studies. [Review] [87 refs]. Cancer.

[B19] Hunt JD, van der Hel OL, McMillan GP, Boffetta P, Brennan P (2005). Renal cell carcinoma in relation to cigarette smoking: meta-analysis of 24 studies.. International Journal of Cancer.

[B20] Brennan P, Bogillot O, Cordier S, Greiser E, Schill W, Vineis P, Lopez-Abente G, Tzonou A, Chang-Claude J, Bolm-Audorff U, Jockel KH, Donato F, Serra C, Wahrendorf J, Hours M, t'Mannetje A, Kogevinas M, Boffetta P (2000). Cigarette smoking and bladder cancer in men: a pooled analysis of 11 case-control studies.. International Journal of Cancer.

[B21] Brennan P, Bogillot O, Greiser E, Chang-Claude J, Wahrendorf J, Cordier S, Jockel KH, Lopez-Abente G, Tzonou A, Vineis P, Donato F, Hours M, Serra C, Bolm-Audorff U, Schill W, Kogevinas M, Boffetta P (2001). The contribution of cigarette smoking to bladder cancer in women (pooled European data).. Cancer Causes & Control.

[B22] Brownson RC, Novotny TE, Perry MC (1993). Cigarette smoking and adult leukemia: a meta-analysis.. Archives of Internal Medicine.

[B23] Law MR, Morris JK, Wald NJ (1997). Environmental tobacco smoke exposure and ischaemic heart disease: an evaluation of the evidence.[see comment]. British Medical Journal.

[B24] Law MR, Wald NJ (2003). Environmental tobacco smoke and ischemic heart disease.. Progress in Cardiovascular Diseases.

